# Development of Simplified Methods for Levitation Force Distribution in Maglev Vehicles Using Frequency Ratio Tests

**DOI:** 10.3390/s24175527

**Published:** 2024-08-26

**Authors:** Wen Ji, Weihua Ma, Shihui Luo, Guofeng Zeng, Feng Ye, Mingbo Liu

**Affiliations:** 1 State Key Laboratory of Rail Transit Vehicle System, Southwest Jiaotong University, Chengdu 610031, China; jiwen@tongji.edu.cn (W.J.); mwh@swjtu.edu.cn (W.M.); 2 Maglev Transportation Engineering R&D Center, Tongji University, Shanghai 201804, China; zengguofeng@tongji.edu.cn (G.Z.); yefeng@tongji.edu.cn (F.Y.); 2210772@tongji.edu.cn (M.L.)

**Keywords:** maglev train, load, bridge, dynamics response, levitation force distribution

## Abstract

Maglev vehicles apply the entire vehicle load uniformly onto bridges through levitation forces. In assessing the dynamic characteristics of the maglev train–bridge coupling system, it is reasonable to simplify the distributed levitation force as a concentrated force. This article theoretically derives the analytical response of bridge dynamics under the action of a single constant force and conducts numerical simulations for a moving single constant force and a series of equally spaced constant forces passing over simply supported beams and two-span continuous beams, respectively. The topic of discussion is the response of bridge dynamics when different degrees of force concentration are involved. High-precision displacement and acceleration sensors were utilized to conduct tests on the Shanghai maglev line to verify the accuracy of the simulation results. The results indicate that when simplifying the distributed levitation force into a concentrated force model, a frequency ratio can be used to analyze the conditions for resonance between the train and the bridge and to calculate the critical speed of the train; the levitation distribution force of a high-speed maglev vehicle can be simplified into four groups of concentrated forces based on the number of levitation frames to achieve sufficient accuracy, with the dynamic response of the bridge being close to that under distributed loads.

## 1. Introduction

Maglev trains have three basic levitation principles, namely, electromagnetic suspension (EMS), electric suspension (EDS) and high-temperature superconducting suspension. Currently, only EMS high-speed (430 km/h) and medium-low speed (110 km/h) maglev trains are being used for commercial purposes [1]. The majority of commercial operational lines are constructed as elevated lines. Since the electromagnetic maglev train is supported on the bridge through the active control of the vehicle, the levitation gap is only 8–10 mm. Therefore, in order to suppress the coupling vibration of the vehicle line, the design of the bridge significantly surpasses the requirements for its structural load-bearing capacity or safety load threshold, which increases the engineering construction cost [2,3]. For example, the measured torsion-to-span ratio of the 25 m simply supported beam of the Changsha maglev line under overworking conditions reaches 1/13,781 [2]; however, according to the industry standard “Code for Design of Medium and Low-Speed Maglev Transportation (CJJ/T 262-2017) [4], the maximum allowable deflection-to-span ratio for medium- and low-speed maglev trains is stipulated as L/3800.

Based on International Union of Railways (UIC) train load diagrams and Chinese train load diagrams, China has established standardized design load diagrams for bridges catering to passenger transportation, intercity transportation, and other specific applications [3], and the technical standard system is very mature. However, there are obvious differences between maglev and wheel–rail vehicle load characteristics in three aspects: (1) Maglev lines lack universality and are designed for a single type of operating vehicle; (2) The levitation force is uniformly applied to the line and has the characteristics of levitation buffering [5], and the wheel–rail force is approximately applied rigidly and concentrated to the track, resulting in significant excitation due to track irregularities; (3) Maglev lines are basically single-line beams or double-line composite beams, while wheel–rail bridges are rich in forms, and the main ones are double-line full-span box girder bridges [6].

Studies have shown that the dynamic response of maglev vehicles to bridges is significantly lower than that of wheel–rail vehicles [7], and the degree of impact of maglev vehicles on bridges with different spans is significantly different [8]. Due to the prominent vehicle–line coupling vibration problem of electromagnetic levitation [9], the bridge stiffness requirements proposed by German maglev experts for the Shanghai high-speed maglev line are conservative, based on the simple treatment of each vehicle as a single moving concentrated load [10], without considering the characteristics of maglev vehicles. Although the design of Shanghai high-speed maglev lines has been proven to be very successful, because it is the first commercial high-speed maglev line in China, for safety considerations, the design of the line has larger margins for strength and deflection, which inevitably leads to an increase in construction costs. Therefore, further consideration of the rationality of the design basis is still of great significance to the future development of maglev transportation. The problem of electromagnetic vehicle–bridge coupling vibration can be roughly divided into three categories [11]:

The first part is the problem of periodic excitation caused by fixed train and vehicle loads acting on elastic bridges. Improper matching of vehicle–bridge structure parameters may cause resonance because the excitation frequency is close to the natural frequency of the track beam. This kind of problem is similar to the traditional coupled vibration problem of wheel–rail vehicle bridges. Generally, the moving load model is simplified according to the structural characteristics of the vehicle, and the influence rule of the train and bridge structure parameters is analyzed [12,13].

The second part is the coupling dynamic action between the vehicle multi-body system, suspension control system, and elastic bridge. Generally, a vehicle–bridge coupling dynamic model is established to simulate the actual state, and complex coupling dynamic models and programs need to be established [14,15].

The third part involves the random vibration of vehicles caused by track irregularities and the analysis of vehicle stationarity. This area of study primarily studies the power spectral density (PSD) of track irregularities applicable to maglev systems, as well as the impact of these irregularities on levitation stability and smoothness [16,17].

This study falls within the scope of research on the first category of maglev vehicle–bridge coupled vibration problems. In terms of the vibration issues related to maglev vehicles and bridges, researchers have conducted extensive studies.

The structural characteristics and dynamic performance of track beams are closely related to the simplified process of moving loads. Existing research has discussed various scenarios of different maglev track beams. 

Article [18] introduced a novel integrated track beam for medium- and low-speed maglev transportation. Through finite element analysis, its strength, stiffness, and natural frequency were compared with those of the existing separate track beams. The results indicated that the new track beam exhibited a 28% increase in bending stiffness, a 19.9% reduction in midspan deflection under static live load, and a 13.6% enhancement in the first vertical natural frequency. Similarly, article [19] validated the good static performance and bearing capacity of the steel–concrete combined part of a maglev separate type track beam through finite element analysis and testing. Additionally, article [20] analyzed the midspan deflection and vibration acceleration of different beam types under coupled conditions. The results showed that simply supported beams have larger midspan deflections, continuous beams have better dynamic performance, and the vibration acceleration of continuous beams is smaller when vehicles pass. The current maglev system can be adapted to various forms of track beam structures, and differences exist in the strength of these different track structures and the extent to which they are influenced by environmental factors.

The track stiffness of the maglev system, a crucial parameter directly influencing the dynamic response, has been extensively examined in related research. Article [21] analyzed the coupled vibration of a maglev train travelling in opposite directions on a large-span steel box girder cable-stayed bridge and found that the levitation gap is sensitive to changes in beam stiffness. It is recommended that a deflection-to-span ratio of 1/3015 be used as the vertical stiffness limit for large-span maglev bridges. Article [22] explored the natural vibration characteristics of tracks with varying stiffness and the dynamic response under current control algorithms, finding that a reduction in stiffness leads to a decrease in the first-order natural frequency of the track beam. Although an increased deflection-to-span ratio exacerbates fluctuations in the levitation gap, it does not affect the tracking performance of the control current within a certain range. Article [23], through the analysis of a vertical coupling model of a maglev vehicle and an elevated bridge, found that both the increase in speed of the maglev train and the reduction in track beam stiffness intensify bridge vibrations, but the amplitude of vibration in high-stiffness beams is smaller; the vertical vibration of the vehicle decreases with the increase in track beam stiffness up to a certain threshold after which the impact is not significant; the condition of the line significantly affects the vehicle’s dynamic response, with poor quality lines increasing the deflection of the elevated bridge and the vertical vibration acceleration of the car body. 

Article [24] found that when bridge stiffness is significantly reduced, dynamic responses intensify, and the acceleration of the car body is more sensitive to stiffness changes than variations in the levitation gap. Vehicle speed and temperature deformation significantly affect the dynamic response of the bridge, while track irregularities and the levitation gap have relatively smaller impacts. However, under specific conditions, they importantly influence the adjustment of the relationship between vehicle dynamic response and bridge stiffness. 

Temperature, as another important influencing factor, was analyzed in related studies. Article [25] focused on the steel box girder track of the Shanghai Lingang low-speed test line, utilizing wavelet decomposition to analyze temperature trends and fluctuations, discovering an approximately linear relationship between temperature difference and deformation. Article [26] investigated the stress and deformation of U-shaped beams under extreme temperature gradients, finding that the maximum stress occurs at the connection between the base plate and the track support frame, while the temperature-induced deformation of the track can reach up to 2.91 mm.

Other factors characterizing maglev systems should also be taken into account when studying the dynamic response of beams. Article [27] employed the LQR method to solve for the feedback gain matrix of the controller, combined with MATLAB simulations, and treating both the track beam and the vehicle body as flexible bodies effectively reduced the mass of the track beam and stabilized the system. In the system’s dynamic response, the changes in the levitation gap and track beam took only 0.8 s, with a levitation gap overshoot of only 2% and a midspan deflection of merely 0.9 mm. Article [28] analyzed the impact of bolt loosening on the modal and dynamic response characteristics of steel track beams at a high-speed maglev maintenance base, concluding that bolt loosening significantly reduced the modal frequency of the track beam, altered the vibration mode, and increased the damping ratio under load. Additionally, bolt loosening exacerbated the resonance phenomenon between the track beam and the train, affecting the symmetry and amplitude of vibrations. Article [29] discussed the effects of track irregularities on system dynamic responses and the stability of train operation. It was found that the coupling between lateral and vertical vibrations of the system is weak, sensitive wavelengths for different degrees of freedom were identified, and the specific mechanisms through which vehicle speed and track irregularities affect dynamic responses were revealed. Article [30] revealed that prestressing significantly enhances the vertical vibration of maglev bridges, and its effect intensifies with increasing vehicle speed.

Based on the full consideration of the basic characteristics of the maglev track, relevant research focuses on optimizing the computational efficiency and accuracy of the models. Article [31] compared the differences in vertical coupled dynamic responses of maglev vehicle/bridge systems between beam element and plate–shell element models, finding that the simplification of the beam element model is reasonable at low speeds, with slightly smaller midspan deflections but similar accelerations. 

On this basis, considering that most existing studies use a single vehicle or an entire train as a centralized load, this calculation method is not sufficiently precise. In this paper, the load of a single maglev train is gradually dispersed to approximate the dispersed load characteristics of maglev systems. Specifically, the load of a single train is decomposed into two to eight pairs of concentrated forces according to the number of sub-suspension groups. The midspan displacement and acceleration responses at various dispersion levels and passing velocities are calculated, taking into account the characteristics of the maglev track. This provides a basis for formulating the load pattern of maglev vehicles.

## 2. Response of Elastic Uniform Beam to Moving Unitary Force

Wheel–rail and maglev vehicles have different loading characteristics on bridges, but the common basis is the response of bridges to moving loads, and the response of single-span simply supported uniform beams to moving loads is the most basic common problem. The resonance caused by vehicle loads is divided into two types. The first type of resonance is caused by a series of evenly distributed loads passing through the bridge, while the second type of resonance is caused by a single load passing through the bridge [32]. The speed of the train with the first resonance tends to be lower than the speed of the train with the second resonance.

### 2.1. Frequency Ratio and Critical Speed

**Figure 1:**
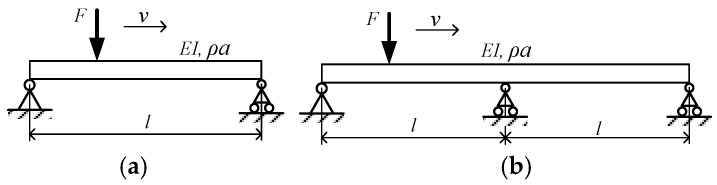
A moving constant force on a uniform beam. (**a**) Single span beam. (**b**) Double span beam.

Figure 1 shows a simply supported beam with a single constant force F crossing the length l at a constant speed of v. It can be proved that this process is equivalent in principle to applying a periodic excitation with a fundamental frequency of $f_{c}=v/l$ to the beam during the duration of (0,v/l), which is called the crossing frequency. The excitation frequency $f_{c}=v/L$ of a series of loads with equal interval *L* to the bridge is called passing frequency to distinguish [33]. In Figure 1, EI, ρ, and a represent the cross-section bending stiffness and mass per unit length of the beam, respectively (ρ is the material density and a is the cross-sectional area). Taking Figure 1a as an example, the dynamic equation of the beam is as follows:

(1) $$EI\frac{\partial^{4}z}{\partial x^{4}}+\rho a\frac{\partial^{2}z}{\partial t^{2}}+c\frac{\partial z}{\partial t}=F\delta (x-vt)$$

where z(x,t) is the displacement of the beam, c is the damping coefficient, and δ is the Dirac delta function.

The response of the beam can be approximately represented by the first *k* modal shapes and generalized coordinates as follows:

(2) $$z(x,t)=\sum_{n=1}^{k}Z_{n}(t)\phi_{n}(x)$$

where $\phi_{n}(x)$ and $Z_{n}(t)$ represent the NTH modal shape function of the beam and its corresponding generalized coordinate, respectively, n=1,2,…k, t∈[0,v/l]. The larger the value of k, the more accurate the results but the greater the computational workload. In the case of undamped free vibration, applying the method of separation of variables to Equation (1) and using the four boundary conditions of a simply supported beam yields the frequency equation for the beam, from which the modal shapes and corresponding frequencies can be determined:

(3) $$\phi_{n}(x)=\sin(\frac{n\pi }{l}\times x),\omega_{n}=\frac{{\left(n\pi \right)}^{2}}{l^{2}}\sqrt{\frac{EI}{\rho a}}$$

Substitute Equation (2) into Equation (1), multiply both sides of the equation by $\phi_{n}(x)$, and then integrate over the entire span length of the bridge to obtain the modal equation for the vertical vibration of the bridge under the generalized coordinate of the NTH mode.

(4) $${\ddot{Z}}_{n}(t)+2\varsigma_{n}\omega_{n}{\dot{Z}}_{n}(t)+\omega_{n}^{2}Z_{n}(t)=\frac{F_{n}(t)}{M_{n}}$$

In the equation, $M_{n}$ and $F_{n}$ represent the generalized mass and generalized force, respectively, while $\varsigma_{n}$ denotes the damping ratio. $\varsigma_{n}=c/(2\rho a\omega_{n})$, $M_{n}=\int_{0}^{l}\rho a\phi_{n}^{2}(x)dx=\frac{\rho al}{2}$, $F_{n}(t)=\int_{0}^{l}[F\delta (x-vt)]\phi_{n}(x)dx≅F\sin(\frac{n\pi }{l}vt)$.

According to vibration theory, when damping and its frequency effects are neglected, the response of Equation (3) to a unit impulse excitation $g(t)=(1/M_{n}\omega_{n})\sin(\omega_{n}t)$ is obtained through convolution integration to find $Z_{n}(t)=\int_{0}^{t}F_{n}(\tau )g(t-\tau )d\tau$, and the final result is:

(5) $$Z_{n}(t)\approx \frac{2F}{\rho al\omega_{n}^{2}[1-{\left(\frac{n\omega_{c}}{2\omega_{n}}\right)}^{2}]}[\sin\frac{n}{2}\omega_{c}t-\frac{n\omega_{c}}{2\omega_{n}}\sin\omega_{n}t]$$

where $\omega_{c}=2\pi f_{c}$. Substitute the obtained modal shape function $\phi_{n}(x)$ and generalized coordinate $Z_{n}(t)$ into Equation (2) and obtain an approximate solution expression for Figure 1a.

(6) $$z(x,t)=\frac{2F}{\rho al}\sum_{n=1}^{k}\frac{\sin\frac{n}{2}\omega_{c}t-\frac{n\omega_{c}}{2\omega_{n}}\sin\omega_{n}t}{\omega_{n}[1-{\left(\frac{n\omega_{c}}{2\omega_{n}}\right)}^{2}]}\times \sin(\frac{n\pi }{l}x)$$

From Equation (5), it can be seen that the passage of a constant force through a simply supported beam is equivalent to imposing a periodic excitation with a frequency of half the modal frequency $f_{c}$. 

In article [10], the ratio of $f_{c}$ to the first-order vibration frequency of the beam $f_{1}$ is defined as the frequency ratio $\eta =f_{c}/f_{1}$. When η=2, it corresponds to the lowest crossing speed that induces the second type of resonance, which is defined as the critical speed $V_{cr}$, apparently:

(7) $$V_{cr}=2lf_{1}=\frac{\pi }{l}\sqrt{EI/\rho a}$$

(8) $$\eta =f_{c}/f_{1}=2v/V_{cr}$$

### 2.2. Numerical Analysis of the Response of an Elastic Uniform Beam

The analysis process for a double-span continuous beam is the same as that for a single-span simply supported beam. Its asymmetric modes and frequencies are identical to the even order modes of a single-span simply supported beam. The frequency equation for its symmetric modes is a transcendental equation, which can only be solved numerically. This section, based on the principles discussed in the previous section and considering the low damping of the beam, uses a 690 kN constant-force concentrated load to represent the maximum weight of a high-speed maglev train according to the general technical conditions for a high-speed maglev train. It calculates the dynamic response of a single constant force crossing single-span and double-span simply supported beams at different frequency ratios using numerical methods. The characteristics of the single-span simply supported beam are shown in Table 1:

Define the bridge displacement response:

(9) $$Z_{m}=Z_{d}/Z_{s}$$

where $Z_{d}$ represents the dynamic deflection at the midspan of the bridge under the action of a moving concentrated load, and $Z_{s}$ denotes the static deflection at the midspan of the bridge when subjected to a concentrated force.

**Figure 2:**
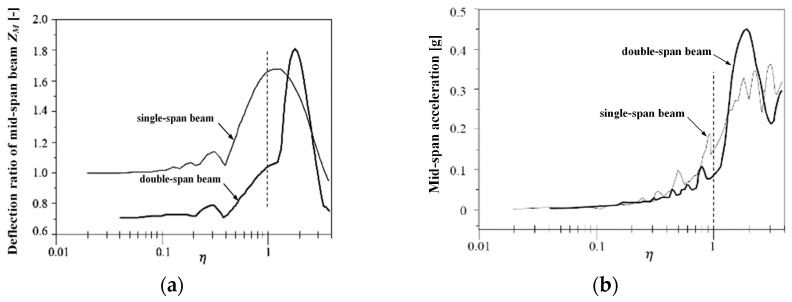
The girder dynamic response under concentrated load: (**a**) Displacement response; (**b**) Acceleration response.

According to the calculation method in Equation (6), the dynamic responses of the two types of bridges under the action of a moving constant force are obtained, as shown in Figure 2, with acceleration response units in g. It can be observed that the dynamic responses of the two types of bridges have the following characteristics: (1) Within the range of η<1.0, the double-span beam performs better than the single-span beam; (2) The acceleration of the double-span beam increases rapidly after η>1.0; (3) The deflection of the single-span beam reaches its peak at η≈1.33; (4) When η=2.0, both the deflection and acceleration of the double-span beam reach their peaks. These characteristics indicate that, regarding the second type of resonance, the double-span beam of this type is suitable for the maximum passing speed of a maglev train of approximately 185 km/h (corresponding to η=1.0). Based on this principle and considering a certain margin, the Shanghai high-speed maglev line selects η≤0.9, requiring that the first-order natural frequency of the double-span simply supported beam should meet $f_{1}\geq 1.1v/l$, corresponding to a maximum design speed of 505 km/h and a span length of 25 m, which gives $f_{1}\geq 6.18\;\mathrm{Hz}$. According to the design documents of the Shanghai maglev line track beam, the vertical first-order natural frequency analyzed using ANSYS 2021 R2 is about 6.77 Hz. The simulation calculations below use this value for the first-order natural frequency.

## 3. Response of Elastic Uniform Beams to Constant Forces at Equal Spacing Series

**Figure 3:**
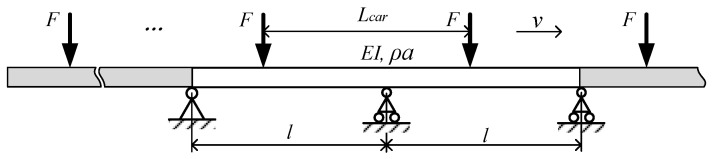
Equal spaces series constant force of double-span continuous girder.

The train is composed of several vehicles, and the single force Fδ(x−vt) on the right side of Equation (1) is replaced by multiple forces. Taking Figure 3 as an example, this parameter can be expressed by: $\sum_{i=1}^{p}F_{i}\delta \left\{x-v[t-(p-1)L_{car}/v]\right\}$, where *p* is the total number of vehicles, and each vehicle is still regarded as a concentrated load. The loading time of the *i*-th force is $t_{i}$ with $t_{i}\in [t_{i0},t_{i0}+2l/v],t_{i0}=(i-1)L_{car}/v$.

**Figure 4:**
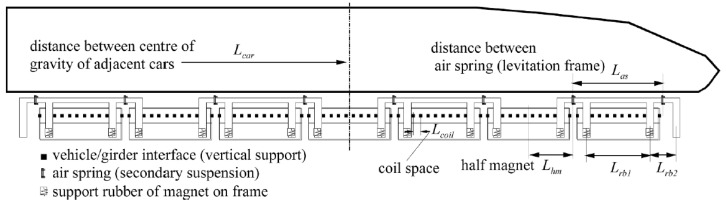
Characteristic length of Shanghai high-speed maglev train.

**Figure 5:**
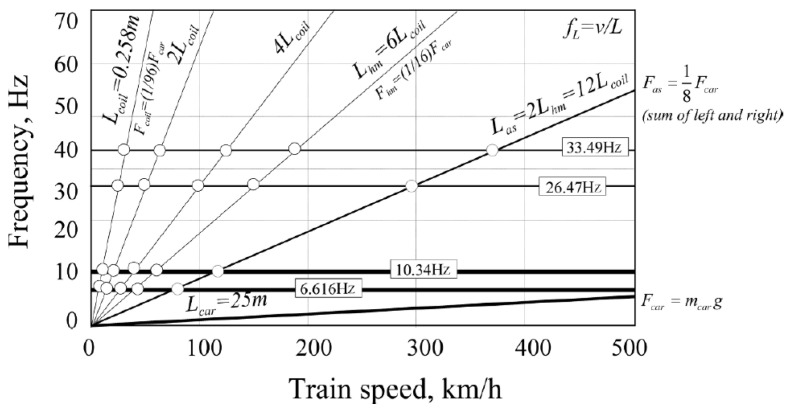
Potential resonance velocity point under moving distributed load.

In this scenario, the first resonance occurs when an equidistant force is equivalent to applying an external excitation to the beam with a period of $f_{p}=v/L$. $L_{car}$ represents the characteristic length of the load between trains. Figure 4 displays various characteristic lengths of Shanghai high-speed maglev trains, including $L_{car}$, $L_{as}$, $L_{hm}$, and $L_{coil}$, corresponding to carriage intervals, secondary spring intervals, semi-electromagnet intervals, and coil intervals (half electromagnet corresponds to six levitation coils), respectively. When these characteristic positions are simplified into concentrated loads, passing frequencies linearly related to speed can be generated as shown in Figure 5. Frequencies that do not vary with velocity represent the first few natural frequencies of the beam, and their intersections correspond to possible resonance velocities. The mass of a single segment vehicle is $m_{car}$, and the load size varies according to different characteristic positions. In the most extreme case, the value of a single segment vehicle is considered as a constant force with a value of $F_{car}=m_{car}g$.

**Figure 6:**
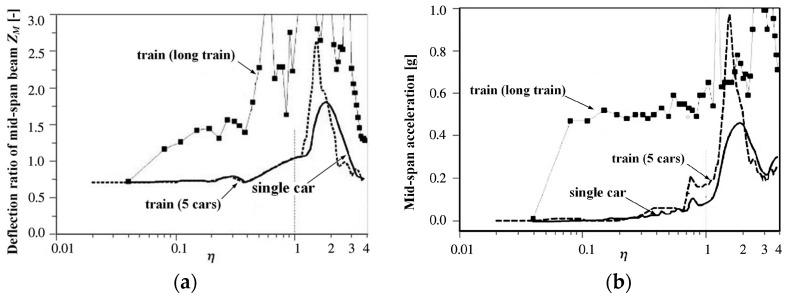
Response of a series of loads under double-span girder: (**a**) Displacement response; (**b**) Acceleration response.

Taking into account that each carriage is considered a constant force (690 kN) in Figure 3, the dynamic response of trains with different formation lengths passing through double-span simply supported beams is calculated and compared with the result obtained by considering concentrated loads. The parameters used for the calculation are identical to those used in the concentrated load calculation. The results are presented in Figure 6, where long train represents a sufficient number of carriages, and the beam vibration is sufficiently amplified by the continuous load.

The findings indicate that with an increase in the number of trains, the deflection and acceleration also rise. At η=1.0 or below, the deflection response of the five trains is basically the same as that of the concentrated load, while the acceleration is slightly higher and there is a slight decrease in the corresponding peak speed. However, for a substantial number of long cars, the beam damping is too small, causing the vibration to fully activate. Based on this result, the allowable train speed is very low, which is not practical and does not align with reality. It is evident that solely applying one concentrated load as the vehicle load on the bridge is unreasonable.

## 4. Simplification of the Concentrated Force of Levitation Distributed Force

Although it is accurate to express the distributed load with the load of the length of the meter, it is usually more convenient to simplify it into a finite number of concentrated forces in the calculation of the train’s entering and leaving process, as well as in the calculation of vehicle dynamics. However, few simplifications create inaccuracy, and too many concentrated forces increase the amount of calculation. Therefore, this section determines the minimum amount of concentrated force required by the bridge’s simplified response to different degrees of concentrated force.

### 4.1. Description of Dispersion Degree

The weight of a rail vehicle is applied to the bogie by at least two pairs of air springs, and the load of each pair of air springs of the wheel–rail vehicle is further applied to the track by two wheelsets. The maglev train body is supported by multiple levitation frames, and the load borne by the air spring of each levitation frame is uniformly applied to the track by the electromagnet.

Assuming that the total gravity load of a vehicle is $F_{car}$, there are *N* pairs of air springs, and they are evenly distributed in the range of the vehicle length $L_{car}$, then the force applied to the track by each pair of air springs is $F_{car}/N$. Considering the number of air springs of the vehicle is N=1,2,…8, it is clear that N=1 is equivalent to the extreme case of simplifying the vehicle to only one concentrated force, N=2 is equivalent to the traditional wheel–rail vehicle, N=3 is equivalent to the BO-BO-BO axle locomotive, N=4−6 is applied in the current low-speed maglev vehicle, and N=8 is equivalent to the Shanghai high-speed maglev vehicle in Figure 4. 

Therefore, the above *N* value can be used as a description of the dispersion degree of the distributed load. The larger *N* is, the higher the dispersion degree is, and the closer it is to the distributed load.

### 4.2. Response of Double-Span Continuous Beam

**Figure 7:**
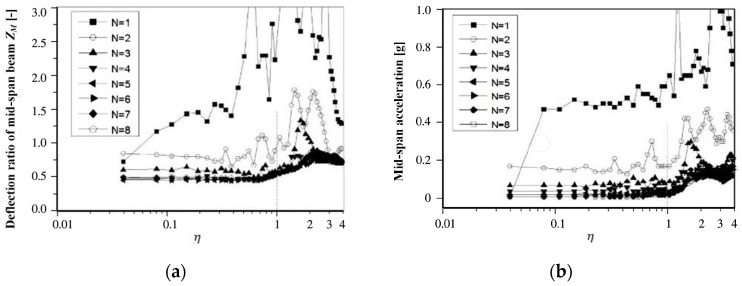
Response of simplifying the distributed forces in different degrees under double-span girder: (**a**) Displacement response; (**b**) Acceleration response.

Using the two-span continuous beam in the above calculation, the gravity load of each vehicle is 690 kN, and the dynamic response of deflection and acceleration in the middle of the first span is calculated when the maglev train with different *N* values and simplified concentrated force passes at different speeds. The results are shown in Figure 7.

From the results of Figure 7, it can be seen that: (1) With the increase of the *N* value, the deflection and acceleration responses of the beam tend to converge to a minimum contour line. (2) The results of N=4,5…8 are basically the same. The characteristic is that the response in the range of η≤0.8 is basically equivalent to quasi-static, and there is a very gentle peak at η=2.0. The peak value of deflection is less than 0.8, and the peak value of acceleration is less than 0.2 g. The characteristics are similar to those in Figure 6, but the magnitude is smaller, indicating that the second resonance is dominant. The results of N=3 have a prominent peak when η is close to 2.0 and an insignificant peak in the range of η<0.9, indicating that the second resonance is dominant, and the first resonance caused by the periodic excitation of the interval force is not obvious. (3) The result of N=2 is similar to that of N=3, but in addition to the slightly lower speed corresponding to the peak, there are other peaks in the range of η<0.9, indicating that the first resonance cannot be ignored. (4) Therefore, it can be considered that the running speed causing the first resonance is lower than that causing the second resonance for rail vehicles. However, for the vehicle structure with N≥4, even if the speed reaches η=2.0, the deflection and acceleration response can meet the requirements. From the Formulas (6) and (7), the critical speed of the double-span simply supported beam used in this case is 377 km/h, and the corresponding running speed of η=2.0 is 377 km/h.

Considering that an air spring of a maglev vehicle corresponds to an electromagnet control unit, and the electromagnet is usually controlled by two controllers each to control a set of coils, it is accurate enough to regard each controller and its coil group as a concentrated force in the formulation of vehicle load diagram and vehicle dynamics analysis.

## 5. Experiments on Site and Discussion

**Figure 8:**
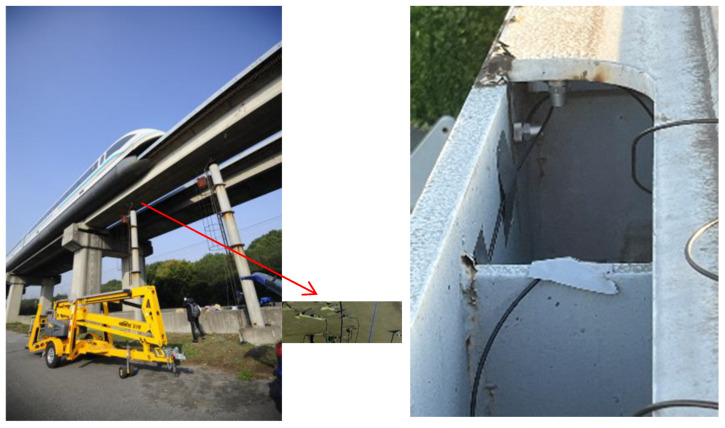
Experiments on site.

To validate the accuracy of the aforementioned simulation results, high-precision laser displacement and acceleration sensors were utilized for testing on the Shanghai maglev demonstration line. The point laser displacement sensor has a measurement range of ±35 mm, a repeatability of 70 um, and a sampling rate of 1 kHz. The acceleration sensor, on the other hand, has a measurement range of ±10 g, a resolution of 0.0004 m/s^2^, a sampling rate of 1 kHz, and a resonance frequency of 8 kHz. These two types of sensors can ensure the precision and accuracy of the measurement data. The steel pipe piles retained from the original tests were used as a benchmark. High-precision laser displacement and acceleration sensors were arranged at the midspan position of the test beam (as shown in Figure 8) to conduct vertical dynamic deflection and acceleration tests.

Firstly, the first-order natural frequency of the 25 m double-span beam on the Shanghai line was measured. An acceleration sensor was attached to the bottom of the test beam. To avoid the influence of vehicle excitation, a vibration acceleration attenuation period after a vehicle passed was selected for spectral analysis to identify the first-order natural frequency.

**Figure 9:**
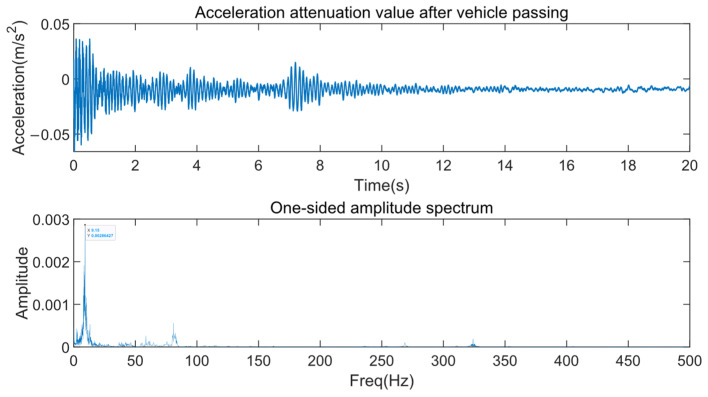
Spectral analysis of the 25 m beam on the Shanghai line.

According to the preceding simulation calculations, to meet the design speed of 505 km/h, it is required that f1 ≥ 6.18 Hz. However, as can be seen from Figure 9, the measured first-order natural frequency is 9.15 Hz, which satisfies the design requirements.

According to the specifications of the Shanghai line system, the static live load for a single horizontal force is 690 kN, which results in a vertical uniform static live load of 27.6 kN/m. Based on the above assumptions, the vertical (static) deflection limit for this beam under a uniform load of 27.6 kN/m is L/4000 = 5.95 mm. The self-weight of one carriage is 18.6 kN/m, and assuming an average of 50 passengers per carriage during testing, estimated at 70 kg per person, this corresponds to a load of 1.4 kN/m. Therefore, for calculation purposes, the vertical uniform static live load q = 18.6 + 1.4 = 20 kN/m is considered. If the assumed actual operational load of 20 kN/m is taken into account, the vertical (static) deflection limit should then be 4.65 mm.

**Figure 10:**
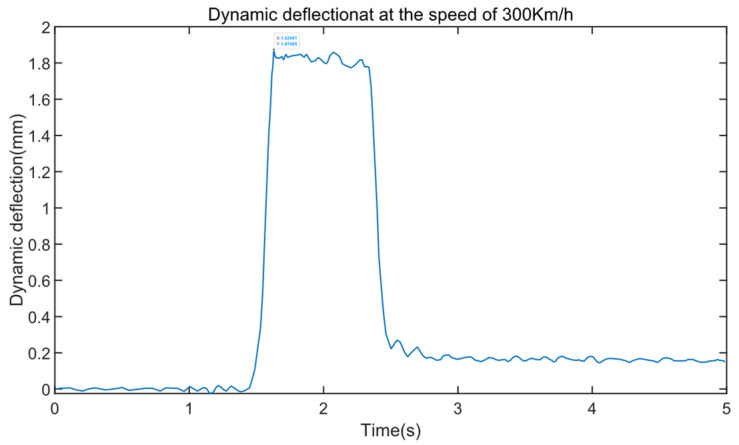
Dynamic deflection at the spend of 300 km/h.

Figure 10 displays the dynamic deflection of the test beam when the train passes through the test beam at a speed of 300 km/h.

From Figure 10, it can be seen that the peak dynamic deflection is approximately 1.875 mm, corresponding to a midspan deflection ratio of $Z_{m}=1.875/4.65=0.403$, at which point the frequency ratio η=0.37. From Figure 7a, it can be observed that when N=1,2,3, the discrepancy between the midspan deflection ratio $Z_{m}$ and the measured values is significant, with smaller *N* values leading to larger discrepancies and less accurate simulation values; when *N* is between 4 and 8, the midspan deflection ratio $Z_{m}$ is closer to the measured values, and as *N* increases, $Z_{m}$ approaches the measured values more closely. 

**Figure 11:**
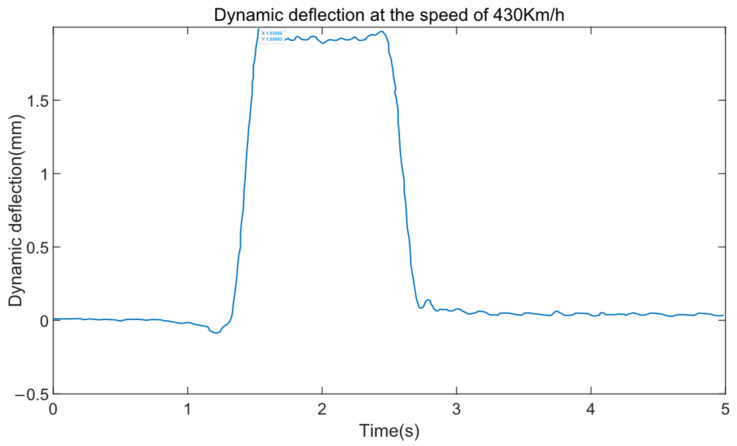
Dynamic deflection at the speed of 430 km/h.

When the train passes through the test beam at a speed of 430 km/h, Figure 11 displays the dynamic deflection of the test beam.

From Figure 11, it can be derived that the peak dynamic deflection is approximately 2.00 mm, corresponding to a midspan deflection ratio $Z_{m}=0.43$, at which point the frequency ratio η is about 0.53. According to Figure 7a, when *N* = 1, 2, 3, there is a significant discrepancy between the midspan deflection ratio $Z_{m}$ and the measured values, with smaller *N* values resulting in larger discrepancies and less-accurate simulation values; the midspan deflection ratios for *N* values between 4 and 8 are all below 0.5, and as *N* increases, $Z_{m}$ approaches the measured values more closely. The Dynamic Amplification Factor (DAF) of the beam deflection increases from 0.403 to approximately 0.43 as the speed increases from 300 km/h to 430 km/h, accompanied by a longer duration of vibration. According to the design specifications of the Shanghai maglev line, the max value of DAF is 1.2. It can be seen that the measured power coefficient value within the current speed range meets the design standard; this also indicates from another aspect that there is a large margin in the design of the guideway beams for the Shanghai high-speed maglev line.

In summary, it can be concluded that when *N* is less than or equal to 3, the simulation calculations differ significantly from the actual test results; when *N* is more than or equal to 4, the simulation calculations are closer to the actual test results. However, as the value of *N* increases, the complexity of the simulation calculations also increases. Therefore, in order to balance the accuracy and complexity of simulation calculations, it is recommended that during simulations, the vehicle load be simplified into four concentrated forces according to the number of levitation frames, and the calculation results can meet the precision requirements.

## 6. Conclusions

This paper studies the dynamic characteristics of the EMS high-speed maglev train–bridge coupling system, discussing the dynamic response when the maglev vehicle distributed loads are simplified into concentrated forces based on theoretical background and dynamic simulations. The following conclusions can be drawn from the study:

1. Simplifying the train as a moving single constant force or a series of equidistant constant forces allows for the analysis of resonance conditions using the frequency ratio η and calculating the train’s critical speeds under different conditions;
2. Through experimental validation, simplifying the vehicle load into four concentrated forces according to the number of levitation frames achieves sufficient computational precision, with the bridge’s response being essentially consistent with that when broken down into more concentrated forces.

## Figures and Tables

**Table 1:** Characteristic parameters of single-span simply supported beam.

Span Length	First-Order Vibration Frequency	Critical Speed	Damping Ratio	Deflection Ratio of a Single-Span Beam
25 m	2.09 Hz	377 Km/h	0.02	1/2500

## Data Availability

The original contributions presented in the study are included in the article, further inquiries can be directed to the corresponding author.
